# Editorial: Affect and cognition in upper echelons' strategic decision making: Empirical and theoretical studies for advancing corporate governance

**DOI:** 10.3389/fpsyg.2022.1081095

**Published:** 2023-01-13

**Authors:** Matteo Cristofaro, Yong J. Bao, Sana Chiu, Ana B. Hernández-Lara, Leticia Perez-Calero

**Affiliations:** ^1^Department of Management and Law, University of Rome ‘Tor Vergata’, Rome, Italy; ^2^Dhillon School of Business, University of Lethbridge, Calgary, AB, Canada; ^3^C.T. Bauer College of Business, University of Houston, Houston, TX, United States; ^4^Department of Business Management, Faculty of Business and Economics, University of Rovira i Virgili, Tarragona, Spain; ^5^Faculty of Business, Universidad Pablo de Olavide, Sevilla, Spain

**Keywords:** behavioral strategy, affect, cognition, decision making, corporate governance

## 1. Introduction to the Research Topic

Since the advent of the bounded rationality concept (Simon, 1947), scholars have been committed to understanding *how organizational agents really make choices*—mainly by adopting social and cognitive psychology lenses. Research over the last 40 years has advanced the investigation of upper echelons' strategic decision-making processes (e.g., Abatecola and Cristofaro, 2020), in which top managers and board directors are regarded as playing a pivotal role in shaping organizational outcomes (Hambrick and Mason, 1984). However, a long-standing limitation in research has been to access the socio-psychological underpinnings of leaders' decision-making, due to the fact that executives are “notoriously unwilling to submit themselves to scholarly poking and probing” (Hambrick, 2007, p. 337).

Recent advances in the space of behavioral strategy show that leaders are different than those postulated by Simon (1947): s/he is no longer affected only by bounded rationality, but s/he is increasingly also pervaded by non-rational forces (Cristofaro, 2017). For example, thanks to the cross-fertilizing advances in neuroscience studies (initiated by Antonio Damasio), the role of emotions—always considered as non-rational forces—has continuously and increasingly gained momentum within decision-making research. The “affect revolution” in research enables the investigation of other important psychological variables considered to be antecedents or consequences of affective states—such as personality traits, mental disorders, beliefs, and spirituality—dismantling, de facto, the “human black box”.

However, what remains largely unknown is the interplay of affective states and cognition (Cristofaro, 2020), considered by some scholars to be two parallel, competitive systems of the human mind (Hodgkinson and Sadler-Smith, 2018). In this regard, our Research Topic for Frontiers in Psychology (2022), entitled “*Affect and cognition in upper echelons' strategic decision making: Empirical and theoretical studies for advancing corporate governance*”, aims to advance this line of inquiry: investigating the role of affective states, cognition, and their interplay in upper echelons' strategic decision making.

## 2. Contributions within the Research Topic

Starting on November 2020, the promotion of the Research Topic through personal contacts with authors, listservs, social media posts, and conference networks raised a total of 25 submissions. Manuscripts aligned with the Call for Papers passed at least two rounds of peer review, always followed by final comments of guest editors. In the end, 43 scholars produced 13 excellent works accepted for publication on this Research Topic. Among them, by considering the first author's institution, 60% of contributions are from China; others are from Italy and the U.S. As of October 17th, 2022, the Research Topic received 24,500 views approximatively, seeing an increasing trend in downloads.

The 13 papers in this issue highlight five crucial research directions for studies on the affect and cognition in upper echelons' strategic decision making: (i) upper echelons' features and personality, (ii) upper echelons' attitude, (iii) upper echelons' identity, (iv) upper echelons' leadership, and (v) domains' cross-fertilization.

**Figure 1:**
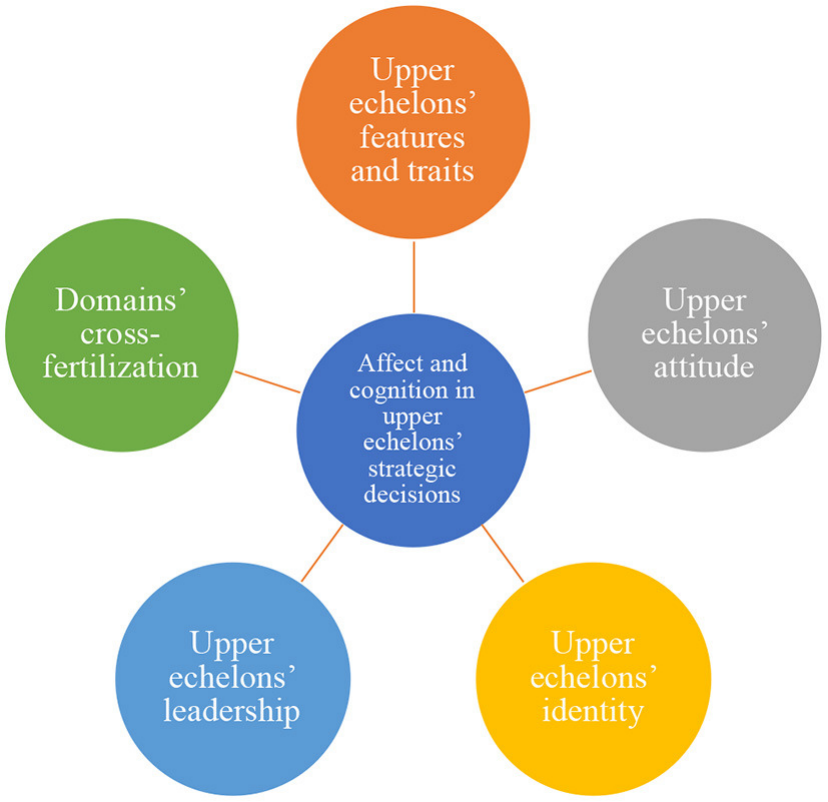
A synthesis of the crucial directions taken for studying affect and cognition in upper echelons' strategic decision making.

We offer some insights emerging from these papers to accompany the readers throughout this editorial endeavor. See Figure 1.

### 2.1. Upper echelons' features and traits

Three studies investigate the innate features and personality of upper echelons and link with cognition, emotions, and decisions. Yang et al., use secondary data from a sample of 64 Chief Executive Officers (CEOs) of listed firms in China for the period 2010–2019, to investigate whether leaders' proactive personality affects Merger and Acquisition (M&A) decisions. They argue that leaders with a proactive personality are more prone to take the initiative to change the external environment. Their study shows that proactive and overconfident CEOs are indeed more inclined toward cross-industry mergers in traditional industries. By contrast, non-proactive CEOs are inclined toward intra-industry mergers. The study by Larsen and Stanley investigates why affect drives decisions in some situations and not others. By comparing New Zealand Prime Minister Jacinda Ardern's and US President Donald Trump's responses to COVID-19, these authors advance that leaders' neurobiological windows of tolerance to affect arousal and their self-regulatory capacity—their capacity to regulate stress and emotions so that these phenomena do not drive resulting decisions—may hold the key to explaining the variation in affect's influence on decision-making. Leaders who prioritize self-care and regulated themselves signal to their followers that self-regulation is a critical aspect of successful performance.

### 2.2. Upper echelons' attitude

Three articles investigates the cognitive influence of attitudes of upper echelons in organizational decisions. Liu et al. collect data on 188 vice CEOs in China regarding their entrepreneurial orientation (i.e., the inclination to emphasize innovation) and firm performance. Results show that vice CEOs' entrepreneurial orientation is positively related to corporate dynamic capabilities and firm performance and that corporate dynamic capabilities mediate the positive relationship between CEO entrepreneurial orientation and firm performance. Similarly, Zheng investigate how the temporal focus at the organizational level affects the investment decisions of venture capital (VC) firms. Using data from 606 VCs with 1,473 VC-year observations of long-term orientation from 2012 to 2019, they show that VCs with a higher level of long-term orientation prefer to invest in less popular industries and ventures in the expansion period. Meanwhile, they are less likely to invest in very new start-ups. Moreover, long-term-oriented VC firms tend to re-invest in start-ups in their portfolios. Finally, by considering observations of 5,352 firms listed in the Shanghai and Shenzhen Stock Exchanges between 2010 and 2019, Jiang et al. investigate whether TMT media exposure can influence Corporate Social Responsibility (CSR) performance. Media exposure acts as an external control mechanism to monitor TMT behavior and that the authors find that a high level of TMT media exposure promotes social responsibility. In addition, the TMT power and political connections negatively moderate the relationship between TMT media exposure and CSR, because these allow TMTs to pursue profit instead.

### 2.3. Upper echelons' identity

Three papers investigate how the identity of the founders or CEOs influences their cognition and decisions. Xiaofei et al. collected 7,491 observations on founders of Chinese private listed companies between 2010 and 2018 to study the effect of the founder of private enterprises on CSR. Their results show that founder-led firms have better CSR performance because of founders have a long-term vision, care about the reputation of the company and take a broad perspective of value-creation activities. Similarly, Ma et al.—by analyzing 2019 IPO data of 635 private companies in the China A-share market through the fuzzy-set qualitative comparative analysis (fsQCA)—found that the presence of a highly educated founder, large firm size, and the absence of risk tolerance as core conditions leads to high innovation input. Yet, the combination of large firm size with having a male, highly educated, highly risk-tolerant founder, coupled with being CEO and having strong political connections, is more conducive to increasing the innovation investment of the firm. Finally, Zhang et al., by using 1,330 firm-year observations of Chinese-listed family firms from 2009 to 2015, investigated the factors that affect the pay dispersion between CEO and non-family managers. This study shows that the presence of non-family CEOs could decrease the pay dispersion between CEO and non-family managers. Empirical evidence also supports that the negative relationship between CEO identity and pay dispersion weakens when CEO tenure increases and the institutional environment matures.

### 2.4. Upper echelons leadership

Two published articles in the Research Topic deal with the role of upper echelons' leadership in their cognition and related decisions. In particular, Xu et al. collected data from 26 supervisors and 304 new-generation employees in a new energy vehicle company in East China to understand how the executives can effectively stimulate the proactive behavior of new-generation employees. Results indicated that (i) distributed leadership is positively related to the proactive behavior of new-generation employees; (ii) idiosyncratic deals and meaningfulness of work mediated the linkage between distributed leadership and new-generation employees' proactive behavior; (iii) idiosyncratic deals and meaningfulness of work play a multistep mediation role between distributed leadership and new generation employees' proactive behavior. Relatedly, Liang et al. investigated leaders' positive implicit followership (LPIF) influence on employees' innovative behavior (EIB) across 389 leaders and their direct employees at 45 large- and medium-sized enterprises in China. LPIF has a significant positive effect on EIB, and leader-member exchange (LMX) and psychological empowerment (PE) have multiple mediation effects on the relationship between LPIF and EIB. When the level of LPIF is high, LMX and PE are also enhanced, promoting the increase in EIB.

### 2.5. Domains' cross-fertilization

A series of studies in the RT pushed cross-fertilization among management and other domains, mainly neuroscience, psychology, and philosophy, to investigate upper echelons' decisions better. The work by Cristofaro et al. provides a systematization of 23 contributions produced on the role of affect and cognition in managerial decision-making by considering the recent cross-fertilization of management studies with the neuroscience domain. Results of the analyses support an emerging “unified” mind processing theory for which the two systems of our mind are not in conflict and for which affective states have a driving role toward cognition. They also strongly recommend using neuroscience methods to support behavioral studies. In line with this latter, Mastrogiorgio et al. investigate how to enhance organizational cognitive memory. They administered a task to 82 employees of a large banking group, comparing the results of a virtual memoryscape (VM; treatment) concerning the mnemonic tool based on the “method of loci” (MoL; control). The VM is not superior to the MoL in the short term, but it is equivalent to 1 week later. Compared to the method of loci, the virtual memoryscape presents the advantages—relevant for organizations—of being collective, controllable, dynamic, and non-manipulable. Finally, Adinolfi and Loia propose an integrated interdisciplinary framework suitable for a rich account of intuition, contemplating how affect and cognition intertwine in the intuitive process, and how intuition scales up from the individual to collective decision-making. In particular, by bridging the psychological, philosophical, and organizational domains, authors conceptualize “intuition as emergence”—a new property that emerges in a decisional space. Intuition, according to that property, *emerges out of self-organizing holistic associations*. In this proposition, the properties of self-organization and emergence are the defining features of intuition.

## 3. Implications for future research

Studies published in the RT advance the research on affect and cognition in upper echelons' strategic decisions. Some studies advanced affect and cognition as two parallel cooperative systems of humans that interact with each other and together relate with the environment in which upper echelons are embedded and from which are influenced by its factors, such as technology. However, to advance this line of research, which finds its natural space within the new field of Behavioral Strategy (Powell et al., 2011), other open research questions still need to be answered, such as: *How do biases scale up from the individual to the collective level? Under what contexts and individual conditions are heuristics beneficial for executives? How do stakeholders' cognition and/or affective states impact those of executives? Do cognitive and affective states play out differently for executives in state or private-owned firms, small and medium-sized enterprises or big firms, family firms, and start-ups? What psychological or cognitive attributes are more likely to influence executives' decisions related to non-financial firm outcomes such as corporate social responsibility or sustainability? What situational factors are likely to moderate this relationship?*

As to help answer the above, we firmly believe that the way traced by some works (Adinolfi and Loia; Cristofaro et al.; Mastrogiorgio et al.) toward the cross-fertilization of (apparently) unrelated domains (e.g., management, psychology, philosophy, neuroscience, etc.) to inform upper echelons' strategic decisions research is the one to beat to open executives' black box and understand the behavior of organizations. This is the main value-added coming from this Research Topic.

## Author contributions

MC contributed to the conception and design of the Editorial and wrote the first draft of the manuscript. All authors contributed to the manuscript revision, read, and approved the submitted version.
